# Nitric Oxide and the Neuroendocrine Control of the Osmotic Stress Response in Teleosts

**DOI:** 10.3390/ijms20030489

**Published:** 2019-01-23

**Authors:** Carla Cioni, Elisa Angiulli, Mattia Toni

**Affiliations:** Department of Biology and Biotechnology “Charles Darwin”, Sapienza University, via A. Borelli 50, 00161 Rome, Italy; carla.cioni@uniroma1.it (C.C.); elisa.angiulli@uniroma1.it (E.A.)

**Keywords:** nitric oxide synthases, teleost, caudal neurosecretory system, hypothalamo-hypophysial system, arginine vasopressin, oxytocin

## Abstract

The involvement of nitric oxide (NO) in the modulation of teleost osmoresponsive circuits is suggested by the facts that NO synthase enzymes are expressed in the neurosecretory systems and may be regulated by osmotic stimuli. The present paper is an overview on the research suggesting a role for NO in the central modulation of hormone release in the hypothalamo-neurohypophysial and the caudal neurosecretory systems of teleosts during the osmotic stress response. Active NOS enzymes are constitutively expressed by the magnocellular and parvocellular hypophysiotropic neurons and the caudal neurosecretory neurons of teleosts. Moreover, their expression may be regulated in response to the osmotic challenge. Available data suggests that the regulatory role of NO appeared early during vertebrate phylogeny and the neuroendocrine modulation by NO is conservative. Nonetheless, NO seems to have opposite effects in fish compared to mammals. Indeed, NO exerts excitatory effects on the electrical activity of the caudal neurosecretory neurons, influencing the amount of peptides released from the urophysis, while it inhibits hormone release from the magnocellular neurons in mammals.

## 1. Introduction

Nitric oxide is a gaseous messenger that is endogenously produced in cells and tissues of all major groups of organisms and acts in a variety of biological processes. Nitric oxide has a short half-life and spreads through the cell membranes, making impossible to store it in vesicles. For these reasons, NO must be generated near its site of action. In animals, NO is enzymatically generated by complex and highly regulated enzymes, known as NO synthases (NOSs), which convert l-arginine into NO plus l-citrulline. Mammalian NOSs are large proteins (molecular weight >130 kDa, 1153–1434 aa), which have a bidomain structure in which the N-terminal oxygenase domain (containing binding sites for heme, tetrahydrobiopterin (BH_4_) and l-arginine) is linked by a calmodulin (CaM)-recognition site to the C-terminal reductase domain that in turn contains binding sites for flavin adenine dinucleotide (FAD), flavin mononucleotide (FMN) and nicotinamide adenine dinucleotide phosphate (NADPH). The known NOS enzymes are homodimers in their active form [1].

Three distinct NOS isoforms have been identified in mammalian tissues, which are products of different genes, with different localization, regulation, and catalytic properties. These isoforms are the so-called neuronal NOS (nNOS, Type I NOS, NOSI or NOS1), inducible NOS (iNOS, Type II, NOSII or NOS2), and endothelial NOS (eNOS, Type III, NOSIII, NOS3). Neuronal NOS was first purified [2] and cloned from neurons but it has also been detected in non-neuronal cells such as skeletal muscle fibers [3]. NOS3 was originally discovered in the endothelial cells [4] and then revealed in several non-endothelial cells including neurons. Both *NOS1* and *NOS3* genes are constitutively expressed and are activated by increases in intracellular Ca^++^ through Ca^++^/CaM binding to the reductase domain of the enzyme. NOS2, is a Ca^++^-independent enzyme first identified in macrophages [5], whose expression can be induced in a wide range of cells and tissues by cytokines and other agents. 

NOS enzymes were first classified as constitutive (NOS1 and NOS3) and inducible (NOS2) forms, but it was then shown that the level of constitutive enzymes can also be modulated by different conditions. Constitutive NOS1 and NOS3 are low output enzymes producing NO involved in physiological signaling. Endothelium-derived NO is a powerful vasodilator agent whereas brain-derived NO mediates neurotransmitter release, synaptic plasticity, neural development, and regeneration [6]. Differently from the constitutive isoforms, NOS2 is a high output enzyme whose activity generates NO having beneficial microbicidal effects or contributing to cellular damage in a variety of immunological disorders, depending on its concentration.

The expression of constitutive NOSs is regulated inside the cell depending on their subcellular localization. NOS3 is bound to the plasma membrane by its myristoylated and palmitoylated N-terminal whereas NOS1 is both soluble and membrane-bound. The association of NOS1 to the membrane is established by the PDZ (PSD-95/Discs Large/ZO-1) domain in its N-terminus anchoring NOS1 to the synaptic membrane via protein to protein interaction with PSD95 (postsynaptic density protein 95) or other PDZ-containing membrane-associated proteins. The PDZ domain of NOS1 interacts with the distrophyn complex in skeletal muscle cells [7] and binds with high affinity to the tight junction membrane proteins claudin-3 and claudin-14 [8].

**Figure 1:**
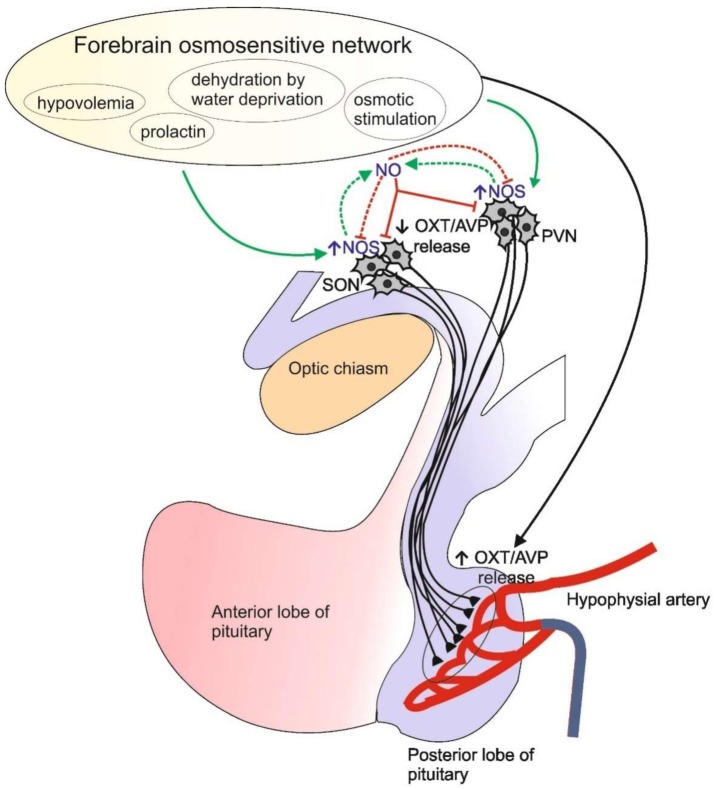
Schematic representation of the HNS showing the NO involvement in the modulation of AVP/OXT release following physiological and stressful stimuli. Green arrows indicate the increase in NO synthases (NOS) synthesis and/or activity; green dashed arrows indicate NO production; red lines indicate reduction of arginine vasopressine/oxytocine (AVP/OXT) release; red dashed lines indicate a putative feedback mechanism reducing NOS activity; black arrow indicates the stimulation of AVP/OXT release [13,14,17].

In mammals, NOS1 expression is regulated by physiological conditions and evidence shows the involvement of NO in the regulation of hypothalamo-neurohypophysial system (HNS) (Figure 1). Various physical, chemical, or biological agents inducing cellular stress result in the up-regulation of NOS1 in the hypothalamus. For instance, immobilization stress induces an increase in *NOS1* mRNA in the rat hypothalamic paraventricular nucleus (PVN) [9]. The expression of *NOS1* can also be modulated by hormones. Indeed, both estradiol and lactation stimulates an increase in *NOS1* mRNA in the rat hypothalamus [10,11]. Suckling during lactation and different stressful stimuli upregulate prolactin (PRL) expression and its release within the PVN in female and male rats [12]. Increased levels of brain or serum PRL enhance nNOS activity in hypothalamic PVN and supraoptic nucleus (SON), and thereby stimulate oxytocine (OXT) and arginine vasopressine (AVP) secretion. PRL-induced release of both neurohormones is prevented by treatment with a selective inhibitor of nNOS, demonstrating the role of NO in modulating OXT and AVP release [13]. 

Although the effects of central nitrergic systems on the hydromineral imbalance are controversial, overall studies show a modulatory role for NO in the release of both AVP and OXT. In both PVN and SON of the rat hypothalamus, the expression of *NOS1* is up-regulated after chronic salt loading [14,15] or water deprivation [16] and NO has as an inhibitory role on AVP and OXT secretion. Recent evidence in hypothalamic and neurohypophyseal explants confirmed that NO negatively modulates the secretion of AVP and OXT induced by the acute extracellular hyperosmolality [14]. NOS inhibition increases hormone release whereas exogenous NO inhibits NOS activity together with the hormone release induced by hyperosmolality. This suggest that NOS1 undergoes some sort of feedback regulation by its product [14] and this mechanism could partially explain the conflicting results obtained in previous studies.

All the available data agree that NO acts as a modulator in the homeostatic balance and sympathetic activity of the hypothalamus of mammals [17]. Both electrophysiological and neuroendocrine evidence confirmed that NO inhibits the activity of magnocellular neurons in the SON and PVN nuclei of the hypothalamus thus influencing the release of AVP and OXT from the neurohypophysis. NO-modulated activity of SON and PVN is under the control of the forebrain osmosensitive network, a neural circuitry of osmosensitive areas receiving information by osmoresponsive elements in the forebrain lamina *terminalis* which, in turn, receives information from central and peripheral osmoreceptors, volume and baroreceptors and circulating angiotensin II [17,18]. The involvement of NO in the modulation of osmoresponsive circuits in the Central Nervous System (CNS) appears to be evolutionarily conserved since it has been demonstrated that NOS enzymes are present in the HNS of teleosts and their gene expression may be regulated by osmotic stimuli. The present paper is a review on the research data demonstrating the modulatory role of NO in the HNS and the caudal neurosecretory system (CNSS), the two neuroendocrine systems controlling osmoregulation in teleosts.

## 2. NOS Enzymes in Teleost Fish: Molecular Insights

NOS enzymes appear to be highly conserved during evolution as NOS homologues have been identified in various organisms including bacteria [19]. The molecular characteristics of the invertebrate NOS have been identified in some taxa [20], such as cnidarians [21], arthropods [22,23], molluscs [24,25,26], and the amphioxus [27].

The phylogeny of the vertebrate NOS isoforms is still relatively unknown and further molecular data are necessary to produce an accurate analysis. Among non-mammalian vertebrates, NADPH diaphorase (NADPHd) histochemichemistry and/or NOS immunocytochemistry have demonstrated that NOS enzymes are expressed in the CNS of fishes [28,29,30,31], amphibians [32,33,34], reptiles [35], and birds [36]. Phylogenetic analysis performed on NOS sequences from the tunicate (*Ciona intestinalis*), the amphioxus (*Branchiostoma floridae*) and some teleost species suggested that a duplication of the ancestral *NOS* gene occurred after the tunicates diverged from the vertebrate lineage [37]. The three NOS isoforms expressed in vertebrates thus evolved from an ancestral form through gene duplication and loss events [20,38]. The diversification of NOS1/3 and NOS2 isoforms presumably occurred following the second whole genome duplication [20] and the presence of two *nos2* genes (*nos2a* and *nos2b*) in some teleost species would derive from the third whole genome duplication event occurred in fish lineages. Donald and collaborators [38] have proposed that the duplication of the *nos1/3* gene that gave rise to the Nos1 and Nos3 isoforms occurred before the actinopterygian-sarcopterygian separation but after the appearance of the chondrichthyan fish lineage. The absence of Nos3 in teleosts would derive from the loss of related genes in this lineage.

In the last three decades several studies have attempted to analyze NOS enzymes in teleosts by immunohistochemistry, NADPHd histochemistry and Western blot analysis (Table 1). More recently, the partial or complete cloning of some *nos* cDNA and the completed fish genome projects have provided new interesting data.

To date, only two NOS enzymes are identified in teleosts: Nos1 and Nos2. cDNA sequences for *nos1* and *nos2* are available in GenBank but no molecular data support the existence of *nos3* in this group of vertebrates. Indeed, whole teleost genomes so far sequenced failed to reveal *nos*3 genes [37,54,59,60,61,62,63]. Differently from teleosts, three *nos* genes have been identified [38] or predicted in the lepisostean spotted gar *Lepisosteus oculatus*: *nos1* (Gene ID: 102699252), *nos2* (Gene ID: 102692649) and *nos3* (Gene ID: 102697373) (https://www.ncbi.nlm.nih.gov/gene/). This evidence indicates that *nos3* gene was present in the bony fish ancestors and it was secondarily lost in the teleost radiation.

### 2.1. NOS1/nNOS

Sequence analysis of nos1 has revealed a high degree of conservation among teleost species (83–100% amino acid identities) and between teleosts and other vertebrates (74–82% amino acid identities with human NOS1, Table S1). Moreover, both partial and full-length cloned sequences in teleosts showed the presence of all the essential domains of the mammalian NOS1. In particular, the domain prediction revealed that Nos1 contain PDZ, oxygenase, FMN-binding, FAD-binding, and NADPH-binding domains in the red drum *Sciaenops ocellatus* and killifish *Fundulus heteroclitus* [37,50]. Phylogenetic analyses in vertebrates revealed that teleost and non-teleost Nos1 belongs to the same cluster while separating from Nos2 [50] (Figure S1). 

Molecular analysis revealed that *nos1* is expressed at high levels in the teleost CNS (brain and spinal cord) as it also happens in mammals (Table 1). Moreover, *nos1* is expressed in not nervous tissues, as gill, heart, kidney, stomach, intestine, liver, spleen, gonads (ovary and testis), anal gland, muscle, skin, thymus, cardinal vein, blood, and in the CNSS (Table 1). Therefore, *nos1* expression is not restricted to neurons in teleosts as well as in mammals. 

The spatial and temporal expression pattern of *nos1* was studied in zebrafish embryo and young larvae: *nos1* was expressed at 16 hours post-fertilization in the hypothalamus and by three days post-fertilization it was present in discrete locations within the CNS as well as in the enteric nervous system [64]. Moreover, the expression of *nos1* mRNA in distinct cell populations of the forebrain was closely followed by the expression in the skin, and subsequently in the medulla and retina, and in distinct cell populations within peripheral organs [65].

### 2.2. NOS2/iNOS

Molecular analyses have revealed that *nos2* gene is not constantly present among teleost species. Indeed, a *nos2* homologue could not be found in the genomes of fugu (*Takifugu rubripes*), tetraodon (*Tetraodon nigroviridis*), stickleback (*Gasterosteus aculeatus*) and medaka (*Oryzias latipes*) [54]. On the other hand, one *nos2* gene was described in the carp (*Cyprinus carpio*; [66]), in the rainbow trout (*Oncorhynchus mykiss*; [67]), in the pacu (*Piaractus mesopotamicus*, [57]) and in many other telost fish (Table 1 and Table S2). Finally, the complete coding sequences of the mRNA of two *nos2* isoforms were described in goldfish (*Carassius auratus*; [54,68]) and two different *nos2* genes were identified in zebrafish (*Danio rerio*, [69]) and channel catfish (*Ictalurus punctatus*, [40]). The absence of *nos2* in some fish species probably derive from the loss of the relative gene in that specific lineage during evolution. It must be considered that teleosts comprise thousands of different species and a specific gene may be present in the genome of some species but absent in others due to gene loss during evolution. For example, it has been shown that alpha synuclein, a “prion-like” protein expressed in the CNS of vertebrates and involved in human neurodegenerative diseases called synucleinopathies [70], is not present in the genome of *Danio rerio* but it is expressed in other teleost species such as *Silurus glanis*, *Esox lucius*, *Oryzias latipes*, *Oreochromis niloticus* and *Takifugu rubripes* [71].

The analysis of Nos2 deduced protein sequences revealed a percentage of amino acid identities of 62–98% among teleosts and of 57–61% between teleosts and human NOS2 (Tables S2 and S3).

In adult teleosts, *nos2* is expressed in the brain, eyes, oral cavity, gill, heart, kidney, intestine, gut, liver, spleen, gonads (ovary and testis), muscles, cardinal vein, and skin (Table 1). Similarly to mammal NOS2, Nos2 is an inducible enzyme involved in the defense of the organism, whose expression is induced in response to bacterial (or lipopolysaccharide) challenge in adult fishes as goldfish (*Carassius auratus*; [53]), rainbow trout (*Oncorhynchus mykiss*; [53,72]), pacu (*Piaractus mesopotamicus*, [57]), carp (*Cyprinus carpio*; [66]) and channel catfish (*Ictalurus punctatus*, [40]). Increased Nos2 protein expression was detected after bacterial infection in spleen, kidney [58] and intestine [56] in turbot (*Psetta maxima*). Nos2 was also shown to participate in the innate immunity in the embryonic carp [73].

In goldfish, two complete *nos2* mRNA coding sequences, *nos2a* and *nos2b*, have been cloned [68]. The two deduced amino acid sequences reveal a percentage of identities of 90% and predicted molecular weights of 127,6 kDa (Nos2a) and 127,8 kDa (Nos2b). Moreover, in goldfish a NOS2-like protein was detected by Western blot in primary cultures of dispersed pituitary cells [74]. This protein immunolabeled with the antibody against murine NOS2 and showed the molecular weight of approximately 120 kDa. It was a constitutively expressed enzyme involved in growth hormone (GH) secretion. 

The presence of two *nos2* genes in zebrafish is a rare example of two complete and different *nos2* genes found in a vertebrate genome [54]. The percentage of amino acid identity between the Nos2a and Nos2b proteins is 68.4% (Table S2). In both enzymes, all the important functional domains identified in NOS2 were observed: the cofactor-binding sites for heme, BH_4_, CaM, FMN, FAD, NADPH ribose, NADPH adenine, and the conserved C-terminal sequence for NADPH binding [54].

The expression of both *nos2a* and *nos2b* strongly increased in the regeneration site, following tail transection in the zebrafish larvae, emphasizing the putative inducible feature of both enzymes [54]. Despite these similarities, other studies have shown interesting differences between Nos2a and Nos2b. The analysis of the Nos2b amino acid sequence revealed the N-terminal myristoylation consensus sequence that is characteristic of *NOS3* in mammals. Interestingly, an N-myristoylation site was also found in carp Nos2 and goldfish Nos2a and Nos2b [54]. Moreover, from the analysis of the basal expression of *nos2* gene in adult zebrafish, *nos2b* appeared to be a more “constitutive” enzyme as it was equally present in all the analyzed tissues, whereas *nos2a* transcripts showed a more restricted pattern of expression [54]. Finally, *in vitro* experiments performed on zebrafish cell lines of hepatocytes and fibroblasts showed that *nos2a* mRNA levels increased following the treatment with pro-inflammatory molecules, whereas *nos2b* mRNA was constitutively expressed. Taken together, data in zebrafish suggests that *nos2b* could exert some of the functions of mammalian NOS3. 

### 2.3. NOS3/eNOS

Whereas the existence of *nos1* and *nos2* genes is proved in teleosts, conflicting information are available for *nos3*. Some studies based on antibodies against mammalian NOS3 initially showed immunoreactivity in fish tissues. In particular, apparent Nos3-immunoreactivity has been found in the spinal cord of tilapia [47], in the vascular endothelium of developing zebrafish [75], in gill tissue in the Atlantic salmon (*Salmo salar*; [76]), in the trout posterior cardinal vein [43], in the heart and endothelial cells of tilapia [77] and in the liver of the Atlantic croaker (*Micropogonias undulatus*) [41]. Moreover, Nos3-like immunoreactivity has been found in the heart tissues of several teleosts (*Anguilla anguilla*, *Chionodraco hamatus*, *Protopterus dolloi*, *Thunnus thynnus thynnus* and *Trematomus bernacchii* [78]). Molecular biology and bioinformatic analyses of sequenced genomes failed to reveal *nos3* gene (*nos3*-like sequences) in teleosts [20,37,54,59,60,61,62,63]. This suggests that the results outlined above may derive from the cross reactivity of the antibodies raised against mammalian NOS3 with different Nos isoforms in teleosts. 

## 3. NO in the Hypothalamo-Neurohypophysial System

### 3.1. Hypophysiotropic Neurons in the Brain of Teleosts

**Figure 2:**
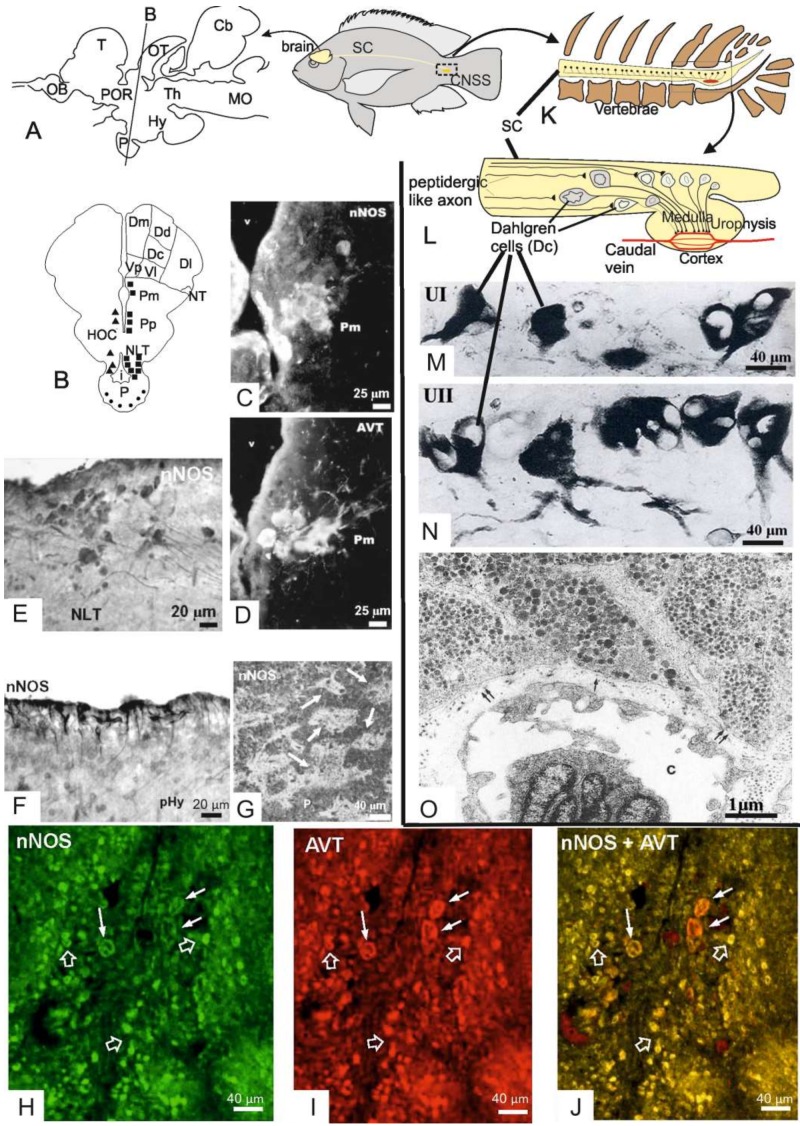
Hypophysiotropic (**A**–**J**) and caudal neurosecretory (**K**–**O**) systems in teleosts. (**A**,**B**): schematic representation of longitudinal (**A**) and transverse (**B**) sections of the brain of adult *Oreochromis niloticus* showing the distribution of Nos1-immunoreactive (square) and NADPHd reactive (triangle) nerve cells. In the pituitary gland (P), dots represent Nos1-ir hypothalamo-hypophysial fibers and pituitary cells. (**C**,**D**): Fluorescence micrographs of consecutive transverse sections through the magnocellular preoptic nucleus (Pm) immunostained for Nos1 (**C**) and AVT (**D**). Note that Nos1 and AVT are colocalized in the same neurons. (**E**): Nos1 immunohistochemistry showing labelled neurons in the lateral zone of the nucleus lateralis tuberi (NLT). (**F**): Nos labelled CSF-contacting neurons in the pHy. (**G**): Fluorescence photomicrograph of the neurointermediate lobe (NIL) of the pituitary gland immunolabelled for Nos1. High magnification showing the distribution of Nos1-ir in the pericapillary areas (arrows). (**H**–**J**) Transverse sections of the Pm and the hypophysis of *O. niloticus* single and double immunolabeled for Nos1 and AVT. Nos1 positive cells are green (490 nm excitation, FITC), AVT-positive cells are red (545 nm excitation, Cy3), while regions of colocalization are lemon green, yellow or orange. Magnocellular neurons immunoreactive for both Nos1 and AVT (arrows). Note the large number of small neurons immunoreactive for Nos1/AVT (open arrows). (**K**,**L**): schematic representation of the terminal tract of the spinal cord showing teleost CNSS characterized by the presence of Dahlgren cells sending their axons to the urophysis. (**M**,**N**): Large Dahlgren cells immunostained respectively for UI and UII in *C. auratus*. (**O**): Electron micrograph of the urophysial cortex. Large granules- (single arrows) and small granules-containing (double arrows) axon terminals are shown. c: capillary. Scale bars are reported. Abbreviations. Cb: cerebellum; CNSS: caudal neurosecretory system; D: *area dorsalis telencephali*; Dc: D *pars centralis*; Dd: D *pars dorsalis*; Dl: D *pars lateralis*; Dm: D *pars medialis*; HOC: horizontal commissure; Hy: hypothalamus; i: infundibulum; MO: medulla oblongata; NLT: *nucleus lateralis tuberis*; NT: *nucleus tenia*; OB: olfactory bulb; OT: optic tectum; P: pituitary gland; pHy: periventricular hypothalamus; Pm: magnocellular preoptic nucleus; POR: preoptic region; Pp: parvocellular preoptic nuclei; SC: spinal cord; T: telencephalon; Th: thalamus; Vl: *area ventralis telencephali*, *pars lateralis*; Vp: *area ventralis telencephali*, *pars postcommissuralis*.

As in other vertebrates, neurons projecting to the pituitary gland are located in the preoptic region (POR), the periventricular and tuberal zones of the hypothalamus (Hy, Figure 2A) of the teleost brain. The periventricular hypothalamus (pHy) includes a large number of cerebrospinal fluid (CSF)-contacting neurons, belonging to the paraventricular organ (PVO), which project to the pituitary gland [79] to regulate the release of several pituitary hormones [80]. The major part of the tuberal hypothalamus is involved in the neuroendocrine regulation of the adenohypophysis that, in teleosts, is directly innervated by lateral tuberal axons. In the teleost POR, magnocellular and parvocellular neurons synthesizing arginine vasotocin (AVT) and isotocin (IT) massively project to the neurohypophysis where the two peptides are released into the blood vessels. 

AVT and IT are orthologous nonapeptides to mammalian AVP and OXT. They display their action in target organs, as brain, gill, kidney, liver, intestine, muscle and heart [81], through specific receptors (AVTr and ITr). In particular, AVT plays crucial roles in the regulation of fish physiology and behavior. Indeed, it is considered the major endocrine mediator of the stress response, the cardiovascular regulation and the osmoregulation, playing similar roles to AVP in tetrapods [82]. Teleost AVT is produced not only by POR neurons innervating the neurohypophysis but also by hypopysiotropic neurons projecting to the corticotrope cells of the adenohypophysis, thus acting as a modulator in the hypothalamo-hypophysial-interrenal axis. Still debated is the AVT expression in pallial and subpallial regions of teleosts [83,84].

The involvement of AVT and IT in the response to osmotic stress is suggested by several studies. Fish transfer to a hyposmotic or to a hyperosmotic environment may stimulate the AVT system in different ways, by modulating AVT gene expression in the brain, by provoking variation in the AVT pituitary content and its plasma concentration or by inducing organ-specific changes in AVT gene expression (for reviews see [85,86]). However, results are often conflicting. For instance, some studies reported significant differences in plasma AVT concentration between fish acclimated at different salt concentration (rainbow trout [87,88], flounder [89,90], rainbow trout [89], gilthead sea bream [91]) while others failed to detect such differences in the same and other species [89,92,93]. However, all studies reported AVT changes during the initial period of acclimation to salinity (see [94,95]). In flounder, plasma AVT concentration transiently decreases when fish are transferred from salt (SW) to fresh (FW) water and this evidence sustains the functional association between AVT and osmoregulation in hypertonic media [93]. According to the most accredited view, AVT contributes to the hydromineral balance by combining antidiuretic effects on the kidney with direct effects on gill water and ion transport [82]. 

The osmotic stress also causes a temporary IT increase in trout plasma [87]. However, studies suggest a minor involvement of IT compared to AVT [88,91,95]. 

### 3.2. NOS Expression in the Hypothalamo-Neurohypophysial System 

According to the present view, NO is involved as modulator in the HNS in fish as in mammals. In the mammalian hypothalamus, the relationships between NOS1, AVP, and OXT are well demonstrated as NOS1 is colocalized with OXT and AVP in the hypothalamic nuclei (SON and PVN) [96,97,98] and specific stimuli or stress conditions determine *NOS1* upregulation in both nuclei and influence the release of OXT and AVP [14,17,99,100,101,102,103]. Recent observations led to the conclusion that, in mammals, NO produced by NOS1 in the SON and PVN negatively regulates the secretion of AVP and OXT and their gene expression (Figure 1) [14,17,103,104,105].

The possible involvement of NO in fish neuroendocrine secretion was first suggested by histochemical studies reporting the presence of NADPHd staining of HNS neurons in teleosts. NADPHd staining is a simple histochemical technique revealing NADPHd activity of NOS enzymes, that catalyze the NADPH-dependent conversion of a soluble tetrazolium salt (NBT) to insoluble visible formazan. Following this reaction, cell bodies and processes of putative NOS-neurons are strongly labelled in a Golgi-like feature. NADPHd is coincident with NOS1 in central and peripheral neurons of mammals [106,107] and NADPHd staining is generally assumed to be an indicator of NOS activity in mammalian tissues. However, NADPHd staining is not always specific for NOS given that several other enzymes can produce formazan by NADPH/NBT [108]. The staining becomes more specific when tissues are fixed with paraformaldehyde, since most of the NOS-unrelated NADPHd activity is abolished after this treatment whereas the NOS1-related NADPHd is insensitive to aldehydes [109].

NADPHd activity related to Nos was first described in the CSF-contacting neurons of the PVO that display an extremely high activity in the rainbow trout [110]. Positive cells and nerve fibers were also detected in the hypophysiotropic nuclei of the tench diencephalon [111], in the PVO and the goldfish hypothalamus [112], in the preoptic region and the adenohypophysis of *Xiphophorus helleri* [113] and in each of the major subdivisions of the catfish hypothalamus [114]. The distribution of NADPHd activity was compared in 15 catfish species and described for the first time in the pituitary gland [114]. Most cells are labelled in the proximal *pars distalis* of the adenohypophysis with few reactive cells in the rostral *pars distalis* (RPD) corresponding to adrenocorticotropic hormone (ACTH)-releasing cells. In the cyprinid *Rhodeus amarus*, NADPHd positive neurons were found in close proximity to gonadotropin-releasing hormone immunoreactive neurons within the preoptic region and tuberal hypothalamus. These findings indicated a direct involvement of NO in the paracrine regulation of hormone release from the adenohypophysis [115].

The distribution of putative NO-producing neurons was specifically investigated in the Atlantic salmon [30] and goldfish brain [31] by combining NADPHd histochemistry with NOS immunohistochemistry using heterologous antibodies against mammalian NOS1. Positive neurons for both Nos1 and NADPHd were found in the salmon brain including the preoptic nuclei. In goldfish, however, magnocellular preoptic neurons were positive for NADPHd but immunonegative for Nos and a similar discrepancy was found in the hypophysiotropic nuclei of the lateral tuberal region. Although data were assumed to be consistent with a hypophysiotropic function of NO, Nos1-ir staining was not detected in the pituitary gland. 

With regard to NADPHd and Nos-immunoreactive (NOS-ir) stainings, it is known that they are not always overlapped in nervous tissues from several species. The two stainings are not coincident in fish and salamander retinas [116], in the tilapia hypothalamus [29], in the rainbow trout [117], chicken [36] and turtle brain [35]. The two stainings are instead almost completely overlapped in the brain of the lizard *Gekko gecko* [118]. Measurements of regional activities of Nos1 and NADPHd confirmed the discrepancy revealed by localization studies in goldfish and brown trout [119]. Moreover, NADPHd staining is not at all representative for Nos in fish olfactory neurons [120]. In tissue sections incubated with specific or less specific NOS inhibitors, NADPHd staining found in the olfactory system was not due to Nos. These findings suggested caution in evaluating NADPHd histochemistry as a reliable marker for Nos1, in non-mammalian brain tissues at least. This should be kept in mind when making conclusions based on NADPHd staining without comparing NADPHd activity with Nos-ir staining or any other specific methodology to detect NOS activity.

The non-specific NADPHd staining found in the preoptic region of goldisfh, chicken and turtle brain was thus interpreted as suggesting that the role of NO as neuroendocrine modulator of magnocellular hypothalamic neurons is restricted to mammalian species [31]. Subsequent research has not confirmed this suggestion. Indeed, our findings in the preoptic-HNS of the Nile tilapia (see below) and analyses on the goldfish brain [121,122] clearly showed that double-labeled NADPHd/Nos neurons are present within both parvocellular and magnocellular subdivisions of the preoptic nuclei. A relatively recent study on the carp *Labeo rohita* [123] confirmed the expression of Nos1 in the preoptic region during post embryonic development, demonstrating that the parvocellular preoptic nucleus is positive for Nos1 at all the developing stages and in adult fish while the magnocellular preoptic nucleus is only Nos-ir in the adult. 

The role of NO as neuroendocrine modulator was also sustained from previous results obtained by NOS immunoelectronmicroscopy and confocal laser-scanning in the brain of the Atlantic salmon [124]. Nos1 molecules were found to be especially abundant in the neurosecretory neurons and nerve processes innervating the pituitary in this species. In the neuropil, Nos1 molecules were associated with the plasma membrane of dendrites and with synaptic vesicles at axon terminals. Direct implication of NO in the hormone release was also demonstrated by *in vitro* studies showing that exogenous NO stimulates GH secretion from the isolated pituitary cells of goldfish [125]. Interestingly, goldfish pituitary cells in vitro express a putative novel Nos2 isoform, which is smaller than Nos1 and constitutively present [74]. The involvement of Nos/NO signaling has been recently established in the luteinizing hormone release from goldfish pituitary cells [126] and this likely involves Nos2 [126,127,128].

### 3.3. NOS Expression in the Preoptic-Hypothalamo-Neurohypophysial System of the Nile tilapia

Research from our group focused on the expression of Nos1 in the neurosecretory neurons of the two neuroendocrine systems of teleosts, the cranial preoptic-HNS (Figure 2A–J) and the CNSS (Figure 2K–O). Research concerned with the CNSS will be reviewed in later paragraphs. As far as the cranial neuroendocrine system is concerned, the presence of Nos activity was first demonstrated by means of the citrulline assay [129] in diencephalon homogenates of the Nile tilapia *Oreochromis niloticus* encompassing the preoptic region, the hypothalamus and the pituitary gland [29]. Our findings revealed a basal expression of NADPH/Ca^++^-dependent Nos activity both in the soluble and in the membrane fractions which share kinetic properties with Nos1 of mammals [29]. Western blot analysis using a human NOS1 polyclonal antiserum crossreacting with fish Nos1 revealed a doublet in both the supernatant and the pellet, which appears to migrate at approximately 150 kDa, a molecular weight corresponding to the single immunoreactive band detected in the homogenates from rat cerebellum used as controls [29]. This finding demonstrated that Nos activity in the tilapia diencephalon may be attributed to a constitutive Nos which is probably similar to the mammalian NOS1. Both NADPHd and NOS immunohistochemistry stained neuronal somata and cell processes within the two major subdivisions of the preoptic regions, the parvocellular (Pp) and the magnocellular preoptic (Pm) nuclei, as well as in the major hypophysiotropic nuclei of the lateral tuberal region of the hypothalamus and in the pituitary gland [29] (Figure 2B,C). 

Although the distribution of Nos staining was similar to that of NADPHd staining, the two reactions were scarcely overlapped since we often failed to detect evidence of NADPHd/Nos colocalization within the same neurons. In general, NADPHd positive neuronal structures were more abundant than Nos1-ir ones. A large number of NADPHd stained and/or Nos-ir neurons were located in the periventricular and subependymal cell layers of the Pp nuclei as well as in the pHy (Figure 2B,C). Some of the positive cells exhibited the typical features of CSF-contacting neurons (Figure 2D). Interestingly, we have for the first time observed large neurons stained by either NADPHd or Nos1 in the Pm. These neurons immunostained for both Nos1 and AVT in consecutive sections (Figure 2C,E) and were unequivocally identified as magnocellular vasotocinergic neurons by double immunofluorescence staining and confocal microscopy [46] (Figure 2H–J). This study has demonstrated that Nos1 and AVT are colocalized in several cell populations of both Pp and Pm nuclei. The pituitary gland was also reactive for Nos1 without being stained for NADPHd. The distribution of immunoreactive granules showed that Nos1 molecules are located in the neurosecretory axon terminals which surround blood vessels of the neurointermediate lobe of the hypophysis (= the neurohypophysis) (Figure 2G) and in glandular cells of the rostral pars distalis of the adenohypophysis. In the neurosecretory terminals of the neurointermediate lobe, Nos1 was colocalized with AVT [46].

Contemporarily, Jadaho and Malz [130] also described Nos1-ir cells in the small-celled periventricular preoptic nucleus (i.e., the periventricular zone along the Pp and Pm nuclei, see [131]), in the periventricular hypothalamus; i.e., *nucleus recessus lateralis* and *nucleus recessus posterioris*, and in the RPD of the adenohypophysis of the catfish, *Synodontis multipunctatus*. However, no evidence was provided for Nos1 staining in the magnocellular neuroendocrine nucleus, the tuberal hypothalamus or the neurohypophysis in this species. Since the small-celled Nos-ir preoptic region is involved in the regulation of pituitary cells [130], results in the catfish also suggest that NO participate in the hypothalamic control of hormone release by the adenohypophysis.

As mentioned above, a certain degree of discrepancy between Nos1-ir and NADPHd staining is present in non-mammalian neurons. We have suggested three possible explanations for this discrepancy. First of all, different subtypes of Nos1 might be expressed, having different sensitivity to fixation. NADPHd activity may be affected by the duration of aldehyde fixation, at least in some regions (i.e., the adenohypophysis) [29]. Therefore, a prolonged time of fixation as that used in our samples (24 h) might have abolished more sensitive Nos activity. Another explanation may be that some Nos1 molecules detected by immunohistochemistry are not enzymatically active, and thus, undetectable by NADPHd histochemistry. The third explanation considers that NADPHd reaction is partially due to Nos-unrelated enzymes. First explanation is sustained by Western blot evidence since an additional band was detected at about 180 kDa in both the supernatant and the pellet of the diencephalon homogenates, which is possibly due to the presence of an additional Nos isoform. Additional Nos1 might be due to the alternative splicing of *nos1* mRNA or to the post-translational modification of Nos1 protein, as occurs for mammalian NOS1. In the cerebellum and the optic tectum of *Salmo salar*, alternatively spliced *nos1* mRNAs have been identified [51] but similar evidence is still lacking for the diencephalon. The distribution of nos1 transcripts in the brain of the adult zebrafish was compared to NADPHd activity and Nos2-ir and these results also suggested the expression of additional Nos1 enzymes in teleosts [39].

To analyze the regulation of *nos1* in the tilapia diencephalon, we have cloned a partial *nos1* cDNA and examined the basal tissue expression pattern and cellular distribution of *nos1* transcripts in the main regions of the CNS [48]. Three different clones encoding a 206-long amino acid sequence were isolated by RT-PCR with degenerate primers based on the conserved CaM-binding domain of known fish Nos1 isoforms. Sequence analysis revealed that the cloned fragment contains the characteristic binding sites of Nos1 proteins, the CaM-binding domain and part of the FMN-binding domain. The comparative analysis showed that the tilapia Nos1 cloned sequence shares 85–99% of amino acid identity with teleost Nos1 (Table S1). Results obtained by RT-PCR showed that the highest expression levels are present in the forebrain followed by the optic tectum, the brainstem, and the spinal cord, whereas *nos1* gene expression is scarce in the cerebellum [48]. Most *nos1*-expressing neurons are localized in the telencephalon and diencephalon, where the hybridization signal is located in several neuronal cell bodies throughout the POR, including the Pm, and in the periventricular layer of the lateral tuberal region of the hypothalamus [48].

The abundance of *nos1* expressing neurons in the diencephalon of Nile tilapia is in agreement with the immunohistochemical findings and provides evidence that NO may act as neuroendocrine modulator in teleosts. Furthermore, these results have provided the basis for further investigation on the regulation of *nos1* gene expression under the salinity challenge.

## 4. Nitric Oxide in the CNSS

### 4.1. The CNSS: Old Structure and New Functions

In addition to the diencephalic system, most fishes possess a CNSS that is located in the caudal segments of the spinal cord (Figure 2K,O). In teleosts, the CNSS is formed by large neurosecretory neurons, the Dahlgren cells, which are distributed in the caudalmost spinal cord (3 to 10 preterminal segments) and a neurohemal organ, the urophysis, contained in the last vertebra. The CNSS has many structural similarities to the HNS. For this reason, the urophysis was initially considered an additional, caudal hypophysis [132]. Dahlgren cells give rise to two descending unmyelinated axon tracts, one for each side, called spino-urophysial tracts, which terminate into the neurohemal organ. The caudal neurosecretory neurons synthesize the urotensins I (UI) (Figure 2M) and II (UII) (Figure 2N), which are packed within dense-cored secretory granules and transported along the axonal processes to the neurosecretory axon terminals (Figure 2O), where they are released into the capillaries of the urophysis and then drained into the general circulation. Non-teleost fishes have a simplified, presumably ancestral, CNSS with Dahlgren cells distributed over an extensive length of the spinal cord and lacking a discrete urophysis. In these fishes, the neurosecretory axons terminate onto capillaries of the ventral spinal cord surface [133].

UI is a 41 amino acid peptide originally discovered in fish CNSS but then identified in the CNS of all vertebrates. It belongs to the family of urocortins (UCN) whose so far identified members, UCN1 (or UI), UCN2, and UCN3, are highly conservative in chordates [134]. UCN peptides are also structurally related to the corticotropin-releasing hormone/factor (CRH/CRF) thus belonging to the CRF superfamily [135]. These neuropeptides together with their receptors and binding proteins constitute the so-called “CRF system” which is involved in the regulation of the stress response by the way of the hypothalamo-pituitary-interrenal axis [136]. Both CRF and UI stimulate the release of the ACTH from fish pituitary cells *in vitro* [137] and the secretion of glucocorticoids (mainly cortisol) from the interrenal cells [138,139]. The CNSS is not the exclusive source of the piscine UI, since both *crf* and *uI* transcripts are expressed in several brain regions in zebrafish and are overlapped in the POR [140]. The CNSS is the major source of CRF in flounder [141]. A positive correlation between *crf* and *uI* mRNA levels was detected in POR and the CNSS of the rainbow trout in response to different kinds of chemical and social stress. However, the regulation of CRF and UI is stressor-, time- and region-specific in teleosts [142].

Beyond the stress response, CRF and UI are involved in a variety of physiological functions, including body fluid homeostasis and osmoregulation [143], vasoregulation, feeding and weight regulation, regulation of locomotor activity, thermoregulation, growth, and reproduction, in several vertebrates including fish (for a review see [135]).

The three *ucn* genes coding for UI, UCN2 and UCN3 have been identified in medaka and their expression was characterized in the brain and spinal cord [144]. Each gene is expressed with a different distribution, suggesting they could serve distinct physiological roles. At the caudal end of the medaka spinal cord, *ucn2* and *ucn3* are not expressed in Dahlgren cells and only few Dahlgren neurons express UI, suggesting a less important role for UI in medaka CNSS compared to other teleost species.

UII is a cyclic peptide (11–15 aa) belonging to the somatostatin-corticostatin superfamily [145]. It was originally purified from the urophysis of *Gillichthys mirabilis* [146] and then isolated from the caudal spinal cord of non-teleost bony fishes [147,148] and lamprey [149] as well as from the brain of teleosts and elasmobranchs [150], the frog brain [151] and the human spinal cord [152]. For a historical overview on the distribution of UII and the expression of UII gene among vertebrates see the reviews by [153] and [154]. 

Several studies have led to the identification of urotensin II-related peptides (URPs) in both teleosts and tetrapods, and today the UII family includes a total of four paralogue genes called *UII*, *URP*, *URP1*, and *URP2* that probably originated through the two rounds of whole genome duplication occurred during early vertebrate evolution [155,156,157].

All the four genes are expressed in teleosts [158] while in tetrapods two genes (*UII* and *URP*) are only present for the loss of the *URP1* and *URP2* genes during evolution [154,158]. In zebrafish two *uII* genes, *uIIa* and *uIIb*, have been identified [159]. The expression study of *URP1* and *URP*2 in this species showed their localization in GABAergic CSF-contacting neurons named Kolmer-Agduhr and suggested that the two genes are not expressed in Dahlgren cells nor in the urophysis [160]. 

Since first studies it emerged that UII is a potent spasmogen and vasoconstrictor of the smooth muscle [161] and it is now clear that UII has a major role in the regulation of the cardiovascular system in both fish and mammals [162]. Moreover, UII is thought to be involved in fish osmoregulation, as UII urophysial content and its plasma concentrations are altered when fish are transferred between FW and SW [93,163]. It appears that UII influences osmoregulation through both indirect spasmogenic activity and direct effects on ion and water fluxes across gills and kidney epithelia [164]. 

Both AVT and IT have been identified and measured in the CNSS [92,165] but it was not established whether AVT and IT are locally produced by Dahlgren cells or transported to the CNSS by axon projections from the brain. 

### 4.2. Morphology and Fine Structure of the Caudal Neurosecretory Neurons in Teleosts 

Morphological features of the CNSS have been investigated in more than 450 species demonstrating interspecies variations in Dahlgren-cell size, their distribution and the urophysial morpho-histology [166]. Readers who are not familiar with this topic can refer to our report giving a detailed description of the CNSS in goldfish [45]. A similar description was performed in zebrafish [167]. 

In goldfish, Dahlgren cells are distributed in two columns within the last three spinal cord segments. Neurosecretory cell bodies decrease in size in the rostro-caudal direction (Figure 2L) with the largest cells (25 ± 9 μm) multipolar or bipolar in shape (Figure 2M,N). Large Dahlgren cells can be distinguished from the ordinary spinal neurons by their large polymorphic nucleus, which is also clearly discernable in the histological preparations. The majority of Dahlgren cells are medium-sized (16 ± 3 μm) and unipolar in shape. Within the caudalmost spinal cord, Dahlgren cells have ovoid nucleus and smaller size (12 ± 3 μm). Small Dahlgren cells represent the single type of neurons found in the spinal cord dorsal to the urophysis, so that they can be identified by position. 

Dahlgren cells are clearly identified when immunostained for UI or UII (Figure 2M,N). They show the cytological features of active protein-secreting cells, i.e., abundant free ribosomes and rough endoplasmic reticulum cisternae, numerous Golgi bodies and a variable number of large dense-cored elementary neurosecretory granules. Unmyelinated Dahlgren axons can be distinguished into large smooth fibers and fine beaded axons provided with Herring body-like varicosities filled with neurosecretory granules. 

Dahlgren cells are enveloped by a neuroglial sheath crossed by myelinated and unmyelinated descending axons establishing synaptic contacts with the Dahlgren cell soma and its proximal processes. Ultrastructural features of the synapses suggest the presence of different types of chemical contacts, presumably related to different neurochemistry. Putative cholinergic, monoaminergic and peptidergic axon terminals have been morphologically identified in the goldfish. Synaptic terminals of similar morphology have also been found in the neuropil of the CNSS of poecilids [168] where both norepinephrine and serotonin afferents were immunohistochemically detected [169,170,171]. Interestingly, ultrastructural observations on goldfish indicated that the small Dahlgren cells receive peptidergic-like innervation by neurosecretory axon terminals which are similar to those found in the urophysis (Figure 2L). Peptidergic-like axon terminals were exclusively found on the cell soma and the initial axon segment of the small Dahlgren cells. These observations suggest that the posterior small Dahlgren cells of goldfish may be functionally synchronized with the anterior cells, at least in part, by local inputs transported from small collaterals of descending neurosecretory axons. Species differences exist in the organization of synaptic inputs to the caudal neurons since the synaptic apparatus has been found to be much less differentiated in zebrafish than in goldfish [167].

### 4.3. UI/UII Production and Cellular Properties of the Caudal Neurosecretory Neurons 

Immunohistochemical evidence showed that UI and UII are not fully colocalized in the Dahlgren cells of all species [172,173,174,175]. For instance, immunoreactive cells for both urotensins are fewer than UII-ir cells in goldfish. Moreover, a majority of UII-ir cells were observed in the CNSS of the European sea bass [176] and comparable results were obtained in zebrafish by double- UI/UII immunolabeling [167]. On the contrary, most Dahlgren cells were UI-ir in flounder whereas few cells immunoreacted for both UI and UII [141,177]. 

Nonetheless, Dahlgren cells are assumed to be able to synthesize both peptides. Results by *in situ* hybridization studies revealed that UI and UII mRNAs are co-expressed in the caudal neurosecretory neurons in carp [178]. Other studies reported few Dahlgren cells expressing both *uI* and *uII* genes in zebrafish [179] and flounder [180]. However, the bulk of electrophysiological studies in flounder sustains that Dahlgren cells belong to a single cell population. Within the cell population, individual cells may express different *uI* and *uII* transcripts or protein levels depending on their activity status. Immunoreactive differences may thus be due to different states of cellular activity in each species. 

Electrophysiological recordings *in vitro* [181] and *in vivo* [182] have initially identified two apparently different types of Dahlgren cells in flounder: the spontaneously active Type 1 and the electrically silent Type 2 cells. However, further studies have shown that individual cells may change their status from silent to active. Therefore, the two cell types represent alternative functional states of the same cell population rather than distinct cell types [180]. Type 1 spontaneous activity includes a bursting activity that is thought to facilitate the release of neuropeptides from the urophysis. This also occurs in the magnocellular AVP neurons of mammals [183] whose bursting activity is known to regulate the release of neurohormone into circulation [184]. Differently from preoptic neurons, the electrical activity of the caudal cells is asynchronous and this probably ensures a continuous rather than pulsatile release of the urophysial peptides [183]. 

### 4.4. Morphology, Fine Structure and UI/UII Content of the Urophysis 

The urophysis is composed by a central medulla filled with the neurosecretory axons of the spino-urophysial tracts, and a cortical region which is occupied by neurosecretory axon terminals contacting blood capillaries. In goldfish, two types of neurosecretory axon terminals were identified due to the different size of their secretory granules (184 ± 20 nm versus 128 ± 20 nm in diameter) (Figure 2O). Two types of neurosecretory axons have also been distinguished in the zebrafish urophysis, namely type A and type B, containing dense-cored granules of different size and electron density. UI and UII immunogold labelings indicated that type A axons accumulate UII within large, less dense-cored granules whereas type B axons accumulate UI in small, dense-cored granules. Some terminals accumulate both UI and UII in the same granules [167]. On analyzing UI/UII localization by gold immunoelectron microscopy we found that the two peptides are colocalized within the same granules in about 60% of the neurosecretory axon terminals in the urophysis of the Nile tilapia. About 35% of the remaining axon terminals immunoreacted for UII only, whilst 5% immunoreacted for UI only [28]. These observations demonstrated that most neurosecretory cells synthesize both UI and UII at detectable levels thus confirming the existence of a single Dahlgren cell population. In addition, our results clearly show that the two urotensins are stored within the same granule. 

### 4.5. Water-Salinity Driven Changes in the CNSS 

Old literature data was conflicting about the effects of environmental salinity on the CNSS morphology and ultrastructure [176,185,186,187,188,189], the aminoacid uptake [190], the innervation [191] and the pattern of UI/UII immunoreactivity of caudal neurons and/or the urophysis. Some data indicated an increase in UI- and/or UII-ir in the Dahlgren cells and the urophysis of fish transferred to low-salinity in comparison to SW-adapted fish [192,193]. Opposite results were reported in other species [194,195]. Furthermore, some studies failed to detect differences between FW- and SW-acclimated fish [175]. These discrepancies have been attributed to: i) species differences and/or ii) different responses of the CNSS to acute or chronic exposure to the osmotic stimulus, given the different experimental procedures followed in the studies. In particular, Larson and Madani [193] first suggested that the CNSS activates adaptive response to osmotic stimuli in the short-term rather than in the long-term and more recent evidence sustain the osmoregulatory role of the CNSS in the acute response to osmotic stress [177,180]. 

### 4.6. NOS Expression and Putative Role of NO in the Modulation of the Teleost CNSS 

We have for the first time investigated the possible involvement of NO in the modulation of Dahlgren cell activity and the urophysial release of urotensins by analyzing the expression of Nos enzymes in the CNSS of the Nile tilapia, *Oreochromis niloticus*. 

In this species, Dahlgren cells are distributed within the last five pre-terminal segments and dorsally to the urophysis. Their size varies from about 50 μm in the anterior region of the CNSS, to less than 10 μm in the terminal spinal cord. About 50% of the neurosecretory cells immunoreacts for both urotensins, whereas the remnants are UI (few) or UIIir only (the majority) [28]. Neurosecretory axons strongly immunoreact for both UI and UII and the same applied to the urophysis. 

Dahlgren cells of the Nile tilapia express Nos1-like proteins. This was demonstrated by combining NADPHd and immunohistochemical stainings for UI and Nos1 that allowed to identify with certainty the neurosecretory Dahlgren cells as Nos-producing neurons [45,47]. In tilapia Dahlgren cells, the NADPHd reaction is completely colocalized with Nos staining. NADPHd granular reaction stained both the cell somata and cytoplasmic processes of caudal neurons suggesting that Nos molecules are synthesized in the cell soma and then transported along the axon to its terminal. Beyond Dahlgren cell somata and neurosecretory axons, the urophysis was also strongly labelled for Nos. In the urophysial cortex, intense labelling was found in the neurosecretory axon terminals surrounding capillaries, where Nos1 molecules were stored within neurosecretory vesicles. 

Biochemical evidence of Nos activity was also provided in the caudal spinal cord. The expression of a constitutive NADPH/Ca^++^/CaM-dependent Nos activity was demonstrated in the caudal spinal cord homogenates of Nile tilapia by the citrulline assay and direct NO production by these homogenates was detected through the conversion assay of oxyhaemoglobin to methemoglobin [196]. Nos activity was present in both the supernatant and the pellet, and Western blot analysis indicated that it could be attributed to Nos-like molecules of similar molecular weight (about 150 kDa) to the NOS1 of mammals. Interestingly, an additional protein band at about 140 kDa was only found in the pellet which was immunostained with a polyclonal antiserum directed against NOS3 of mammals [196]. Since *nos3* has not been identified in fishes, this evidence is probably due to the cross-reactivity of Nos1 with heterologous antibodies. The intracellular distribution of Nos1 molecules was analyzed for NOSI, NOSI/UI and NOSII/UII by single and double gold immunoelectron microscopy in order to compare the distribution of Nos1-like molecules with that of the urophysial peptides [197]. The subcellular localization of fish Nos1 correlates well with the biochemical data. Indeed, the greatest amount of Nos1 is cytosolic in the neuronal perikarya of the Dahlgren cells. In the neurosecretory axons and axon terminals, Nos1-like molecules are instead associated with the vesicle membrane or the dense core of peptidergic granules, which are the storage sites for UI/UII peptides [28]. Double labelings provided evidence that Nos1 may be colocalized with either UI or UII within the neurosecretory granules. The subcellular distribution of Nos1 in fish CNSS corresponds to that observed in mammalian neurons [198].

The association of fish Nos1 with the granule membrane may be explained by assuming that a protein-protein interaction occurs between soluble Nos1 molecules and some vesicular membrane-associated protein that is responsible for targeting Nos1 to secretory granules. The same occurs for mammalian NOS1 whose association to the plasma membrane is mediated by PDZ-PDZ interactions with membrane proteins such as the PSD-95, PSD-93 or syntrophin [7]. In fish Nos1, the PDZ-domain appears to be conserved and the association of Nos1 with secretory vesicles might ensure a locally elevated concentration of Nos molecules at the neurosecretory axon terminals.

On the basis of our results, we have hypothesized a functional role for NO in the regulation of peptide release from the urophysis. Indeed, Nos1 contained in the neurosecretory terminals might become activated to produce nitric oxide following Ca^++^ influx due to the electrical activity of the excited neurosecretory neurons. NO could then act at as an anterograde messenger that locally modulates the release of urotensins from the axon terminals. Given the high membrane permeability to NO, this gaseous modulator might extend its influence to the neighboring axon terminals even though they are not electrically activated. The resulting local flow of NO could thus represent a short-range mechanism to synchronize the discharge of peptides from neighboring terminals in response to appropriate physiological stimulation. In addition, NO produced at the neurosecretory terminals could modulate the blood flow in the urophysis thus exerting indirect effects on the peptide release. 

## 5. Putative Role of NO in the Neuroendocrine Modulation of the Osmotic Stress Response

According to the studies performed on mammals, NO is involved in the modulation of the electrical activity of the neurosecretory neurons. It has been shown that *nos1* expression increases in the HNS of mammals during osmotic manipulation and this increase has been attributed to the increasing demand for NO modulation of the magnocellular neurons [17]. Moreover, electrophysiological evidence demonstrated that NO inhibits the electrical activity of magnocellular neurons in mammals [199]. 

Given that Nos enzymes are expressed by the neuroendocrine cells regulating osmoregulative processes in teleosts, it could be hypothesized a role for NO in the modulation of the neuroendocrine secretion in response to osmotic stress.

To validate this hypothesis, we have analyzed the effect of the acute hyperosmotic stress on the expression of *nos1* and Nos activity in the POR and the hypothalamus of the Mozambique tilapia, *O. mossambicus* [42], which express Nos enzymes [200]. Fish were exposed to 70% SW and the response to salinity was evaluated in the first eight hours following the salinity change by semi-quantitative RT-PCR. We have observed that *nos1* expression was rapidly and transiently up-regulated in response to the increasing salinity. In fact, the expression level increased four hours after the salinity change and returned to levels corresponding to those of FW-acclimated fish within the eight hours of hyperosmotic challenge [42]. The time course of the *nos1* up-regulation corresponds to the temporary increase in plasma osmolality measured in the Mozambique tilapia after transfer to 70% artificial seawater [201]. Moreover, 4 h after the SW transfer, NADPHd stained neurons were increased in number in the POR. This increase was not generalized but specifically regarded the magno- and giganto- cellular neurons as the number of NADPHd parvocellular neurons was not significantly changed. 

These findings have been interpreted as suggesting that the endogenous Nos1 activity of magno-cellular AVT-neurons is stimulated by the acute hyperosmotic stress with a resulting increase in NO production. Since it has been shown that hypothalamic AVT mRNA expression, AVT hypophysial content and AVT plasma levels change during the acute response to hyperosmotic challenge [94], our findings prompted us to speculate that in euryhaline teleosts, NO may modulate AVT release in the acute response to hyperosmotic stress, in a similar way to mammals and birds [17,202].

Electrophysiological evidence also indicated that NO may act as modulator of the electrical activity of neuroendocrine cells in response to osmotic challenge. In these studies, the nitrergic modulation was investigated by intracellular and extracellular recordings from Dahlgren cells in isolated CNSS preparations from SW- and FW-acclimated flounder, superfused with the NO-donor, sodium nitroprusside [203]. Results showed that NO is strongly excitatory, inducing bursting activity in previously quiescent neurons. However, no difference was found in the percentage of bursting cells between CNSS preparations from SW- and FW-acclimated fish, though burst duration was significantly shorter in SW Dahlgren cells. On the basis of this data, authors have hypothesized that Nos1 is up-regulated during SW adaptation with a resulting increase in NO production which might be responsible for the increased UII-secreting activity of Dahlgren cells in SW-acclimated flounder [163]. Exhaustive evidence for a role of NO in modulating the electrical activity of Dahlgren cells was then provided in the same species [204]. NO donors influence the electrical activity of Dahlgren cells, both by increasing firing rate and by the recruitment of previously silent cells. Quantitative RT-PCR analyses suggested that NO modulation is involved in short-term adaptation to SW as *nos1* gene is over-expressed at eight hours following the fish transfer from FW to SW and returned to basal level within 24 h. 

## 6. Conclusions 

Evidence reviewed here strongly indicate that NO is involved in the modulation of hormone release during the osmotic stress response in teleosts. Indeed, active NOS enzymes are constitutively expressed by both neurosecretory hypophysiotropic and caudal neurons and their expression may be regulated in response to the osmotic challenge. Since NO is a known modulator of hormone release in the OXT and AVP neurons of mammals, present knowledge in teleosts suggests that its role appeared early during vertebrate phylogeny. Thus, the role of NO as neuroendocrine modulator is conservative. Nonetheless, NO seems to have opposite effects in mammals and fish, as it inhibits hormone release from the magnocellular neurons of mammals whilst it exerts excitatory effects on the electrical activity of fish caudal neurons, which is related to the amount of peptides released from the urophysis. 

Multiple evidence in tilapia and flounder demonstrates that *nos1* gene expression is transiently up-regulated in the first hours following hyperosmotic stress. These findings are in line with evidence in mammals which demonstrated the short-term up-regulation of *NOS1* [15,205] and the increased number of NOS1-ir neurons [206] in the hypothalamic neurosecretory nuclei after salt loading. 

NO may thus act in the acute response of fish to the increased salinity by modulating the expression of specific genes (AVT, *uI*, *uII*) involved in the regulation of multiple osmoregulative processes. Furthermore, NO may act as a local synchronizer of the electrical activity of the diffuse Dahlgren cell population and as a local modulator of hormone release from the neurosecretory axon terminals in both the hypophysis and the urophysis.

## Figures and Tables

**Table 1:** Nos enzyme expression detected in different tissues of adult teleosts fish. Black and grey cells indicate respectively the detected and undetected Gene (G)/ Protein (P) expression in the related tissue/organ.

	NOS1																		NOS2												
Specie	*D. rerio*	*F. heteroclitus*	*I. punctatus*	*M. undulatus*		*O. mossambicus*	*O. mykiss*		*O. niloticus*							*S. maximus*	*S. ocellatus*	*S. salar*	*A. anguilla*	*C. auratus*	*C. carpio*	*D. rerio* (*nos2a*)		*D. rerio* (*nos2b*)	*I. punctatus* (*nos2b1*)	*I. punctatus* (*nos2b2*)	*O. mykiss*		*P. maxima*	*P. mesopotamicus*	*S. maximus*
Gene/Protein Expression	G	GP	G	P	G	GP	GP	G	P	P	P	P	G	P	G	G	G	G	P	G	P	G	G	G	G	G	G	GP	P	G	P
**Tissue/organ**																															
**Brain**																															
**Eyes**																															
**Spinal cord**																															
**Oral cavity**																															
**Operculum membrane**																															
**Gill**																															
**Heart**																															
**Head kidney**																															
**Posterior kidney**																															
**Trunk kidney**																															
**Kidney**																															
**Stomach**																															
**Intestine**																															
**Gut**																															
**Liver**																															
**Spleen**																															
**Ovary**																															
**Testis**																															
**Muscle**																															
**Cardinal vein**																															
**Blood**																															
**Lymphocytes**																															
**CNSS**																															
**Skin**																															
**Thymus**																															
**Reference**	[39]	[37]	[40]	[41]	[41]	[42]	[43]	[44]	[28]	[45]	[46]	[47]	[48]	[29]	[48]	[49]	[50]	[51]	[52]	[53]	[52]	[54]	[54]	[54]	[40]	[40]	[55]	[44]	[56]	[57]	[58]

## Supplementary Materials

Supplementary materials can be found at http://www.mdpi.com/1422-0067/20/3/489/s1.

## Abbreviations

ACTH	Adrenocorticotropic hormone
AVP	Arginine vasopressin
AVT	Arginine vasotocin
AVTr	AVT receptor
BH4	Tetrahydrobiopterin
CaM	Calmodulin
CNS	Central nervous system
CNSS	Caudal neurosecretory system
CRH	Corticotropin-releasing hormone
CRF	Corticotropin-releasing factor
CSF	Cerebrospinal fluid
FAD	Flavin adenine dinucleotide
FMN	Flavin mononucleotide
FW	Fresh water
GH	Growth hormone
HNS	Hypothalamo-neurohypophysial system
iNOS	Inducible NOS
ir	Immunoreactive
IT	Isotocin
ITr	Isotocin receptor
NADPH	Nicotinamide adenine dinucleotide phosphate
NADPHd	NADPH diaphorase
NBT	Nitro blue tetrazolium chloride
NIL	Neurointermediate lobe
nNOS	Neuronal NOS
NO	Nitric oxide;
NOS/Nos	NO synthase
NO synthases	NOSs
OXT	Oxytocin
PDZ	PSD-95/Discs Large/ZO-1
pHy	Periventricular hypothalamus
Pm	Magnocellular preoptic nucleus
POR	Preoptic region
Pp	Parvocellular preoptic nuclei
PRL	Prolactin
PSD95	Postsynaptic density protein 95
PVO	Paraventricular organ
RPD	Rostral pars distalis
SON	Supraoptic nucleus
SW	Salt water
UCN	Urocortins
UI	Urotensin I
UII	Urotensin II
URPs	UII-related peptides
