# Supplementary figures and images for: Nitric Oxide and the Neuroendocrine Control of the Osmotic Stress Response in Teleosts
Source: Int J Mol Sci. 2019 Jan 23;20(3):489. doi: 10.3390/ijms20030489 (PMC6386840; doi:10.3390/ijms20030489)

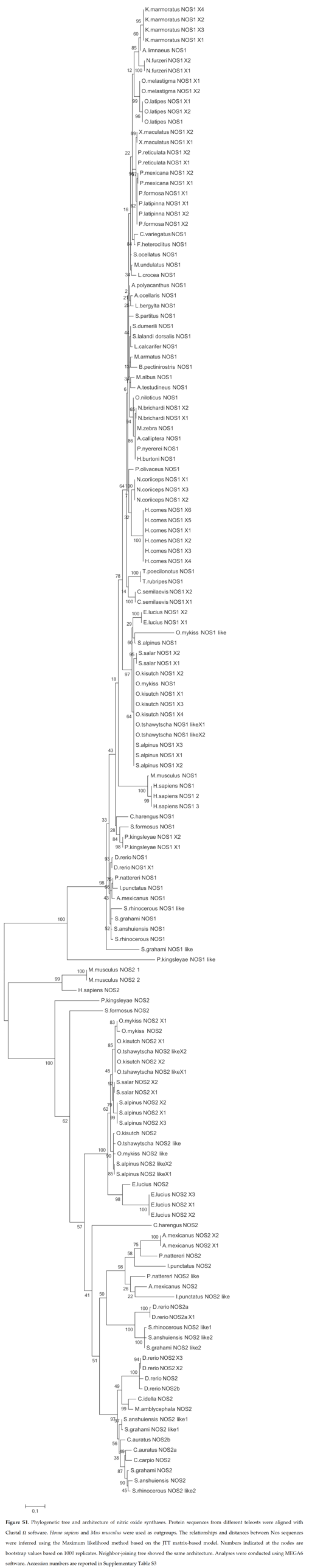

Supplement: Supplementary file 1 [file ijms-20-00489-s001.zip › Figure S1.png]
